# Absorption, Metabolism and Excretion of Cranberry (Poly)phenols in Humans: A Dose Response Study and Assessment of Inter-Individual Variability

**DOI:** 10.3390/nu9030268

**Published:** 2017-03-11

**Authors:** Rodrigo P. Feliciano, Charlotte E. Mills, Geoffrey Istas, Christian Heiss, Ana Rodriguez-Mateos

**Affiliations:** 1 Division of Cardiology, Pulmonology and Vascular Medicine, Medical Faculty, University of Düsseldorf, Moorenstrasse 5, Düsseldorf 40225, Germany; rodrigopedrofeliciano@gmail.com (R.P.F.); geoffrey.istas@kcl.ac.uk (G.I.); Christian.Heiss@med.uni-duesseldorf.de (C.H.); 2 Division of Diabetes and Nutritional Sciences, Faculty of Life Sciences and Medicine, King’s College London, Franklin Wilkins Building, 150 Stamford Street, London SE1 9NH, UK; charlotte.1.mills@kcl.ac.uk

**Keywords:** cranberry, bioavailability, dose-response, kinetics, inter-individual variability

## Abstract

The beneficial health effects of cranberries have been attributed to their (poly)phenol content. Recent studies have investigated the absorption, metabolism and excretion of cranberry (poly)phenols; however, little is known about whether they follow a dose response in vivo at different levels of intake. An acute double-blind randomized controlled trial in 10 healthy men with cranberry juices containing 409, 787, 1238, 1534 and 1910 mg total (poly)phenols was performed. Blood and urine were analyzed by UPLC-Q-TOF-MS. Sixty metabolites were identified in plasma and urine including cinnamic acids, dihydrocinnamic, flavonols, benzoic acids, phenylacetic acids, benzaldehydes, valerolactones, hippuric acids, catechols, and pyrogallols. Total plasma, but not excreted urinary (poly)phenol metabolites, exhibited a linear dose response (*r*^2^ = 0.74, *p <* 0.05), driven by caffeic acid 4-*O*-ß-d-glucuronide, quercetin-3-*O*-ß-d-glucuronide, ferulic acid 4-*O*-ß-d-glucuronide, 2,5-dihydroxybenzoic acid, 2,4-dihydroxybenzoic acid, ferulic acid, caffeic acid 3-*O*-ß-d-glucuronide, sinapic acid, ferulic acid 4-*O*-sulfate, 3-hydroxybenzoic acid, syringic acid, vanillic acid-4-*O*-sulfate, (4*R*)-5-(3′-hydroxyphenyl)-γ-valerolactone-4′-*O*-sulfate, 4-methylgallic acid-3-*O*-sulfate, and isoferulic acid 3-*O*-sulfate (all *r*^2^ ≥ 0.89, *p <* 0.05). Inter-individual variability of the plasma metabolite concentration was broad and dependent on the metabolite. Herein, we show that specific plasma (poly)phenol metabolites are linearly related to the amount of (poly)phenols consumed in cranberry juice. The large inter-individual variation in metabolite profile may be due to variations in the gut microbiome.

## 1. Introduction

Several clinical trials have showed the beneficial effects of cranberry intake on human health [1,2,3,4,5,6,7]. However, while in vitro studies have shown dose-response effects of cranberry (poly)phenols on the inhibition of microbial invasion of gut and bladder epithelial cells [8,9] and in the cytotoxicity of ovarian cancer cells [10], very few human dose-response studies have been conducted.

Howell et al. reported an ex vivo dose-dependent effect on *E. coli* anti-adhesion in urine after cranberry consumption [11], and more recently, cranberry powder intake has demonstrated a dose-dependent effect on reducing urinary tract symptoms in men [12]. However, dose-response studies with cranberry interventions that report improvements in human health not related to the urinary tract are scarce. We recently reported a dose-dependent enhancement in endothelial function after cranberry juice intake [13], while other works did not reveal any improvement in endothelial function or blood pressure after different doses of quercetin-3-*O*-glucoside, a flavonoid present in cranberries, although an increase in quercetin circulating metabolites was reported [14].

To the best of our knowledge, few works have investigated plasma (poly)phenol dose-response profiles after intake of (poly)phenol-rich foods. A statistically significant increase was seen in the maximum concentration (C_max_) and area under the curve (AUC) of pelargonidin-glucuronide, pelargonidin-3-*O*-glucoside and cyanidin-3-*O*-glucoside after increasing amounts of strawberry powder, which were correlated with insulin response and inflammatory markers [15]. However, not all (poly)phenols seem to follow a linear dose response. After green tea consumption, the sum of catechins detected in plasma did not follow a linear dose response, despite the fact that (-)-epicatechin and (-)-epigallocatechin gallate increased their plasma concentration proportionally with the dosage intake [16]. These results suggested an apparent saturation mechanism which was also reported for some plasma (poly)phenols after wild blueberry consumption [17].

We have previously reported that improvements in endothelial function after cranberry consumption correlated with plasma levels of phenolic metabolites, mostly deriving from phase II metabolism [13]. In this work, we evaluate whether the plasma and urine levels of 60 phenolic metabolites found after consumption of cranberry juice with five different (poly)phenol concentrations follow a dose response and we calculate the extent of their inter-individual variability.

## 2. Materials and Methods 

### 2.1. Chemicals

Sulfates and glucuronides of (poly)phenol standards were obtained from Toronto Research Chemicals (Toronto, ON, Canada). Kaempferol-3-*O*-ß-d-glucuronide was obtained from Extrasynthese (Genay, France). The 1-methylpyrogallol-*O*-sulfate, 2-methylpyrogallol-*O*-sulfate, 4-methylcatechol-*O*-sulfate, 4-methylgallic-3-*O*-sulfate, catechol-*O*-sulfate, pyrogallol-*O*-1-sulfate, pyrogallol-*O*-2-sulfate and vanillic acid-4-*O*-sulfate were kindly provided by Dr Claudia Nunes dos Santos and Dr Rita Ventura, and their synthesis has been described elsewhere [18]. The 2-, 3- and 4-hydroxyhippuric acids were purchased from Enamine (Kiev, Ukraine). Remaining compounds were obtained from Sigma-Aldrich Co. (Steinheim, Germany). Acetic acid was from Carl Roth (Karlsruhe, Germany) and Oasis HLB µElution plates (2 mg sorbent per well, 30 µm) were from Waters (Eschborn, Germany). Milli-Q system (Merck KGaA, Darmstadt, Germany) ultrapure water was used. Unless otherwise stated, all chemicals and reagents were obtained from Sigma-Aldrich Co. (Steinheim, Germany).

### 2.2. Human Study 

The study protocol has been described previously [13]. In brief, ten healthy young individuals with ages between 18 and 35 years old participated in a cross-over randomized double-blinded control-matched clinical trial which was registered under the NIH [19] ClinicalTrials.gov website (NCT02517775). All procedures involving human subjects were approved by the University of Düsseldorf Research Ethics Committee (ref: 14-012) and all procedures were performed following the Declaration of Helsinki guidelines. Volunteers were instructed to follow a low-polyphenol diet and exclude berry intake for 24 h prior to each visit. Volunteers were fasted for at least 12 h prior to each intervention to reduce background diet interference as much as possible. During the study day, the same low (poly)phenol meal was giving to all volunteers together with the test drink, and no other food or drink was allowed until after 8 h post-consumption, except for water ad libitum. Participants consumed cranberry juice containing 409, 787, 1238, 1534 and 1910 mg total (poly)phenols (TP) in a random order with 1 week wash out. On the study day whole blood was collected at 0, 1, 2, 4, 6, 8 and 24 h and urine was collected 0–8 and 8–24 h after intake. Plasma was obtained by centrifuging whole-blood collected in EDTA-containing vacutainers for 15 min at 1800 g for at 4 °C; plasma samples were spiked with 2% formic acid. Ascorbic acid was added to the urine containers (3.75 g/2 L) and acidification with formic acid until pH 2.5 was achieved. Urine containers were kept in opaque cool bags with ice blocks. Plasma and urine aliquots were stored at −80 °C until analysis.

### 2.3. (Poly)phenol Analysis in Plasma

Plasma was analyzed with a previous validated µ-SPE coupled with UPLC-Q-TOF-MS that allowed for high-throughput [20]. Briefly, 600 µL of plasma were centrifuged at 15,000× *g* for 15 min at 4 °C and 350 µL of the supernatant was diluted (1:1) with phosphoric acid 4% and spiked with taxifolin (50 nM) as an internal standard. 600 µL were loaded on a 96 well µ-SPE HLB plate, washed with 200 µL of water and 200 µL of 0.2% acetic acid and finally eluted with 60 µL of methanol. Extracted and concentrated plasma samples were analyzed with an Agilent 6550 iFunnel Accurate-Mass Quadrupole Time-of-Flight Mass Spectrometer (Q-TOF MS) (Agilent, Waldbronn, Germany) after separation on a 1290 Infinity UPLC system (Agilent, Waldbronn, Germany) using a Zorbax Eclipse Plus RRHD column 2.1 mm × 50 mm, 1.8 µm (Agilent, Waldbronn, Germany). The mobile phase consisted of 0.1% HCOOH (solvent A) and acetonitrile with 0.1% HCOOH (solvent B). The elution profile (flow rate of 0.4 mL/min) started at 1% solvent B and increased to 10% after 5 min, to 25% at 8 min and to 99% at 9.1 min. The percentage of solvent B was held constant for 0.9 min. The Q-TO-MS parameters were as follows: Negative mode, gas temperature 150 °C, gas flow 20 L/min, nebulizer 25 psig, sheath gas temperature 350 °C, sheath gas flow 12 L/min and Vcap 3000 V. Data were analyzed and processed using Mass Hunter Workstation Quantitative and Qualitative Analysis software (version B.06.00, Agilent, Waldbronn, Germany).

### 2.4. Data Treatment and Statistical Analysis

The maximum plasma concentration (C_max_) and time needed to reach it (T_max_) and AUC of time vs. concentration (calculated using a trapezium method) were calculated using the PKSolver add-in software for Microsoft Excel [21]. Linear regression analysis was performed between (poly)phenol amount from each juice and the average AUC, giving a regression equation and *r*^2^ values. All statistical analysis was performed using GraphPad Prism V6. Significance was defined as *p <* 0.05.

## 3. Results

### 3.1. Plasma Kinetics of Cranberry (Poly)phenols

A total of 60 compounds in plasma were identified and quantified post-consumption of cranberry juice containing 409, 787, 1238, 1534 and 1910 mg total (poly)phenols, as we have previously reported for the juice containing 787 mg of (poly)phenols [22]. These included cinnamic acids, dihydrocinnamic, flavonols, benzoic acids, phenylacetic acids, benzaldehydes, valerolactones, hippuric acids, catechols, and pyrogallols.

Full details of the plasma kinetic profiles can be found in Supplementary Tables S1 and S2. In summary, plasma concentrations varied from low nM to mid µM. The maximum concentration in plasma (C_max_) ranged from 1–31,269 nM (after 409 mg TP), 1–263,152 nM (after 787 mg TP), 2–115,012 nM (after 1238 mg TP), 12–267,355 nM (after 1534 mg TP) and 5–390,606 nM (after 1910 mg TP). The highest C_max_ for juices was achieved for 3-(4-hydroxyphenyl) propionic acid, followed by hippuric acid and catechol-*O*-sulfate.

The time to reach C_max_ (T_max_) varied depending on the metabolite and depending on the amount of (poly)phenols in the juice (Supplementary Figure S1). The level of TP in cranberry juice seemed to impact the T_max_ of individual metabolites, for example the T_max_ for 3-(4-hydroxyphenyl) propionic acid varied between 7.2 h (409 mg TP juice) and 13.8 h (787 mg TP juice).

The AUC of the plasma concentration against the time plot was calculated for each metabolite between 0 and 24 h and was between 2–3,119,972 nM*h (caffeic acid and 3-(4-hydroxyphenyl) propionic acid at 409 mg TP), 1–2,274,346 nM*h (caffeic acid and hippuric acid at 787 mg TP), 2–1,609,708 nM*h (caffeic acid and 3-(4-hydroxyphenyl) propionic acid at 1238 mg TP), 1–390,606 nM*h (caffeic acid and 3-(4-hydroxyphenyl) propionic acid 1534 mg TP) and 5–2,467,256 nM*h (chlorogenic acid and 3-(4-hydroxyphenyl) propionic acid at 1910 mg TP). The compounds with a higher AUC for each dose were hippuric acid > catechol-*O*-sulfate > 2,3,dihydrobenzoic acid > phenylacetic acid.

### 3.2. Plasma Dose Response 

**Figure 1:**
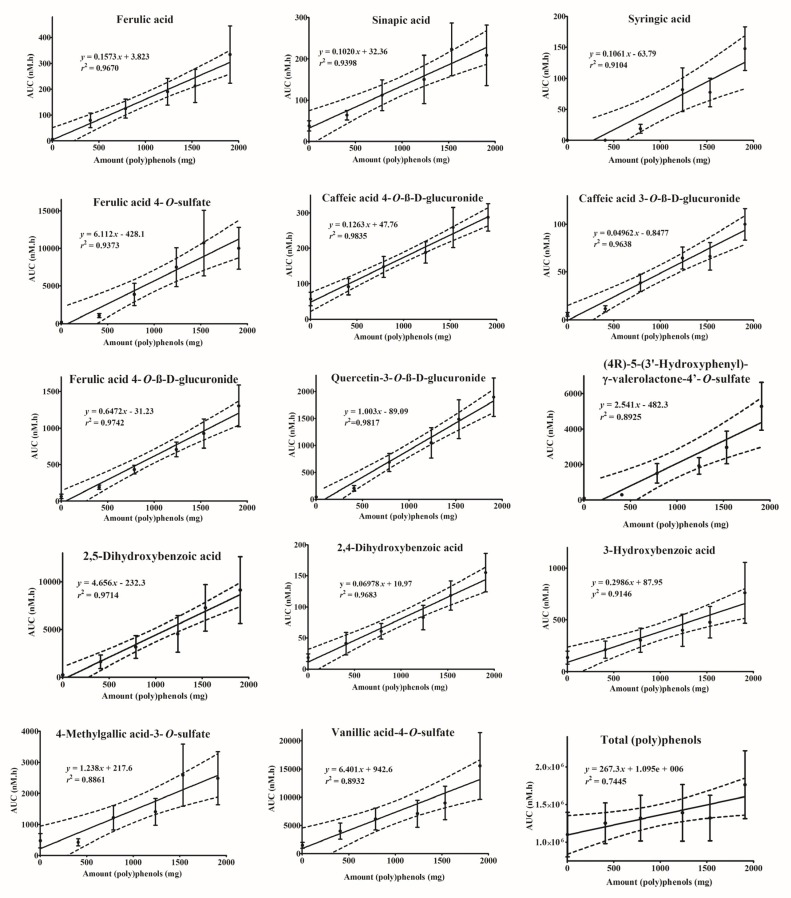
Linear regression analysis between (poly)phenol amount (mg) contained in the cranberry juice interventions and the average area under the curve (AUC (nM*h)) and maximum concentration (C_max_) (nM). Data presented as mean ± standard error of the mean.

The linear dose response for each plasma metabolite was assessed and of the 60 identified metabolites, 14 (poly)phenol metabolites displayed a linear dose-response curve (*r*^2^ ≥ 0.89) in plasma (Figure 1) with slopes significantly different from zero (*p <* 0.05): caffeic acid 4-*O*-ß-d-glucuronide and quercetin-3-*O*-ß-d-glucuronide displayed *r*^2^ > 0.98, while ferulic acid 4-*O*-ß-d-glucuronide, 2,5-dihydroxybenzoic acid, 2,4-dihydroxybenzoic acid, ferulic acid, caffeic acid 3-*O*-ß-d-glucuronide, sinapic acid, ferulic acid 4-*O*-sulfate, 3-hydroxybenzoic acid and syringic acid exhibited *r*^2^ > 0.90. Vanillic acid-4-*O*-sulfate, (4*R*)-5-(3′-hydroxyphenyl)-γ-valerolactone-4′-*O*-sulfate, 4-methylgallic acid-3-*O*-sulfate and isoferulic acid 3-*O*-sulfate had *r*^2^ values of almost 0.90. The sum of the (poly)phenol metabolites also exhibited a modest but significant (*p* > 0.05) correlation of *r*^2^ = 0.74 when plotted vs. (poly)phenol content in the interventions.

Linear regression analysis was performed between the TP level in the intervention and the AUC of each class of compounds. The AUC for flavonols produced the highest *r*^2^ (0.97, *p <* 0.001), followed by the valerolactones (*r*^2^ = 0.89, *p <* 0.005), benzoic acids (*r*^2^ = 0.85, *p <* 0.01), cinnamic acids (*r*^2^ = 0.72, *p <* 0.05), phenylacetic acids (*r*^2^ = 0.71, *p <* 0.05), benzaldehydes (*r*^2^ = 0.65, *p <* 0.05); hippuric acids, pyrogallols, and catechols did not show a linear dose response.

### 3.3. Inter-Individual Variability

**Figure 2:**
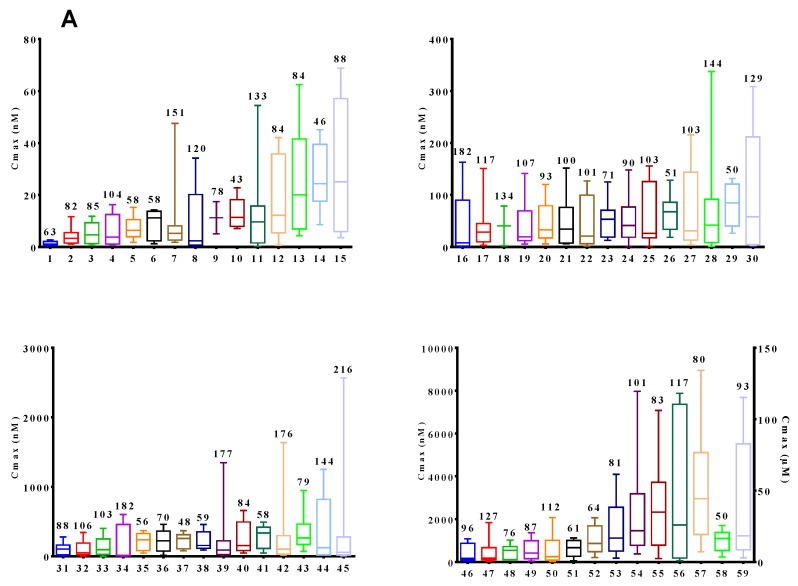

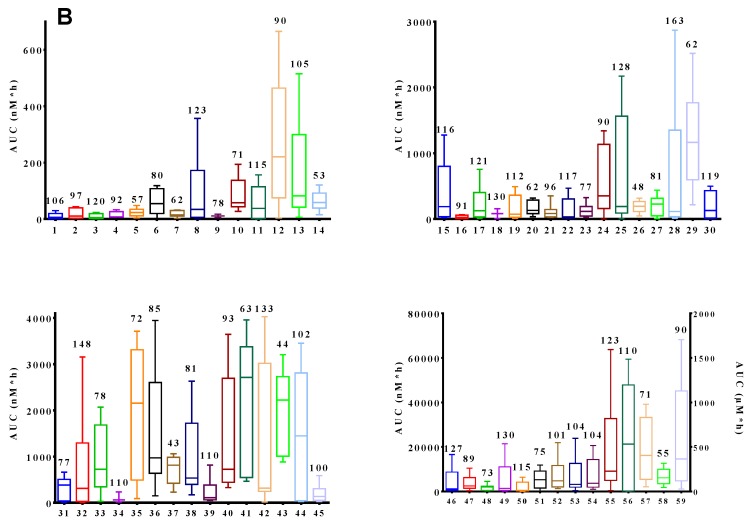
Box and whisker plot of (a) Maximal concentration in plasma (Cmax) and (b) Area under the curve (AUC) of the plasma concentration over time of individual plasma metabolites after 787 mg total (poly)phenol from cranberry juice. Data are presented as median plus upper and minus lower quartiles. Whiskers represent the maximum and minimum values obtained. The coefficient of variation expressed as % is indicated above of the box and whisker plot for each metabolite. Legend. 1. *o*-coumaric acid, 2. 2-hydroxyhippuric acid, 3. Chlorogenic acid, 4. Dihydro Isoferulic acid 3-*O*-ß-d-Glucuronide, 5. 3,4-dihydroxybenzaldehyde, 6. Kaempferol-3-glucuronide, 7. Caffeic acid, 8. 3-hydroxyhippuric acid, 9. 2,4-dihydroxybenzoic acid, 10. Dihydro ferulic acid 4-*O*-sulfate, 11. m-coumaric acid, 12. Syringic acid, 13. kaempferol, 14. Homovanillic acid sulfate, 15. Protocatechuic acid, 16. t-cinnamic acid, 17. sinapic acid, 18. Caffeic Acid 3-ß-d-Glucuronide, 19. 3-hydroxybenzoic acid, 20. Dihydroferulic acid, 21. Pyrogallol-*O*-1-sulfate, 22. 4-hydroxybenzoic acid, 23. *trans*-ferulic acid, 24. 2-Methylpyrogallol-*O*-sulfate, 25. Isoferulic acid 3-*O*-sulfate, 26. 4-hydroxybenzaldehyde, 27. dihydrocaffeic acid, 28. 1-Methylpyrogallol-*O*-sulfate, 29. DihydroIsoferulic acid 3-*O*-sulfate, 30. Dihydro Ferulic Acid 4-*O*-ß-d-Glucuronide, 31. Dihydro Caffeic Acid 3-*O*-Sulfate, 32. Caffeic Acid 4-ß-d-Glucuronide, 33. Dihydro Caffeic Acid 3-*O*-ß-d-Glucuronide, 34. p-coumaric acid, 35. Isovanillic acid, 36. 2-hydroxybenzoic acid, 37. Pyrogallol-*O*-2-sulfate, 38. 4-hydroxyhippuric acid, 39. 4-Methylgallic-3-*O*-sulfate, 40. Quercetin-glucuronide, 41. 2,5-dihydroxybenzoic acid, 42. Homovanillic acid, 43. 3-hydroxyphenyl acetic acid, 44. (4R)-5-(3′,4′-Dihydroxyphenyl)-gamma-valerolactone-4′-*O*-sulfate, 45. vanillic acid, 46. Ferulic acid 4-*O*-glucuronide, 47. 3,4-dihydroxyphenyl acetic acid, 48. 4-hydroxyphenyl acetic acid, 49. benzoic acid, 50. Isoferulic Acid 3-*O*-ß-d-Glucuronide, 51. 4-Methylcatechol-*O*-sulfate, 52. Vanillic acid-4-*O*-sulfate, 53. isoferulic acid, 54. Ferulic Acid 4-*O*-Sulfate, 55. phenylacetic acid, 56. 2,3-dihydroxybenzoic acid, 57. Alfa-hydroxyhippuric acid, 58. Catechol-*O*-sulfate, 59. Hippuric acid.

The coefficient of variation (CV) expressed as a percentage was calculated for C_max_ and AUC for each individual plasma metabolite after consumption of juice containing 787 mg TP; the %CV was used to assess the inter-individual variability after consumption of the juice (Figure 2). The CV of the AUC for the total (sum of all 60) metabolites was 53% and the CV for C_max_ was 51%. The CV for the C_max_ varied between 43% for dihydroferulic acid 4-*O*-sulfate and 216% for vanillic acid. The CV for the AUC varied between 48% for 4-hydroxybenzaldehyde and 163% for 1-methylpyrogallol-*O*-sulfate.

### 3.4. Urinary Recovery

**Figure 3:**
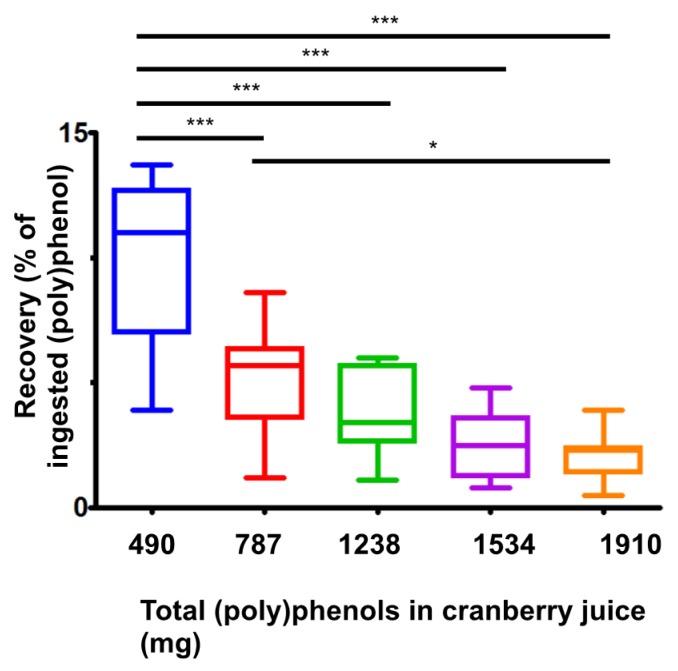
Percentage urinary recovery of (poly)phenol metabolites after consumption of cranberry juice with varying amounts of total (poly)phenols. Data are presented as median plus upper and minus lower quartiles. Whiskers represent the maximum and minimum values obtained. Means were compared using one-way ANOVA and Tukey’s test (* *p <* 0.05 and *** *p <* 0.001).

The same 60 (poly)phenol metabolites quantified in plasma were quantified in urine. Urinary excretion of (poly)phenol metabolites over the 24 h period post cranberry juice consumption is shown in Figure 3. Between 38 and 48 mg total urinary metabolites were excreted post-consumption of the five juices with varying TP content. The mean percentage recovery varied between 10.1% for the juice containing 406 mg TP down to 2.1% for the juice containing the highest TP content (1910 mg). The juice containing the least TP (409 mg) had a higher percentage excretion compared to all the other juices (*p <* 0.001).

A positive, linear dose response for total excretion over 24 h of each urinary metabolite was assessed and of the 60 identified metabolites, 12 (poly)phenol metabolites displayed a positive linear dose-response curve (*r*^2^ ≥ 0.66) in urine with slopes significantly different from zero (*p <* 0.05); they were: 2,3-dihydrobenzoic acid (*r*^2^ = 0.79), 2,4-dihydrobenzoic acid (*r*^2^ = 0.77), dihydrocaffeic-3-*O*-sulfate (*r*^2^ = 0.66), ferulic-*O*-4-sulfate (*r*^2^ = 0.89), *o*-courmaric acid (*r*^2^ = 0.72), quercetin-3-*O*-ß-d-glucuronide (*r*^2^ = 0.89), 2,5-dihydroxybenzoic acid (*r*^2^ = 0.80), chlorogenic acid (*r*^2^ = 0.72), p-coumaric acid (*r*^2^ = 0.90), sinapic acid (*r*^2^ = 0.85), benzoic acid (*r*^2^ = 0.99), isoferulic acid (*r*^2^ = 0.74). The sum of (poly)phenol metabolites did not exhibit a linear dose response (*r*^2^ = 0.32, *p* = 0.25) when plotted vs. (poly)phenol content in the interventions.

## 4. Discussion

In this study, we quantified 60 poly(phenol) metabolites in plasma and urine after consumption of cranberry juice containing five differing amounts of (poly)phenols. Other than previous work by our group where the same 60 metabolites were quantified in a single concentration of cranberry juice [22], this is a much larger number of metabolites than usually reported after cranberry juice consumption [23,24,25]. Further, here we present quantities of glucuronidated and sulfated metabolites in addition to the methylated and native metabolites, unlike many of the previous works looking at cranberry (poly)phenol metabolites. This study demonstrates that the most abundant plasma (poly)phenol metabolites post-ingestion of cranberry juice are 3-(4-hydroxyphenyl) propionic acid, hippuric acid, and catechol-*O*-sulfate; this was regardless of the amount of TP in the drink. Despite quantifying a comparatively large number of metabolites in this investigation, it should be noted that this is by no means the entirety of the cranberry (poly)phenol metabolome. Further understanding of the metabolic pathway and synthesis of further metabolites as standards is essential to fully elucidate this.

This study investigated the relationship between the amount of (poly)phenols in cranberry juice and the levels of the corresponding metabolites in plasma. These data show that selected plasma (poly)phenol metabolites correlate with the amount of (poly)phenols ingested with cranberry juice, showing a linear dose response. However interestingly, this does not apply to all (poly)phenol metabolites. Although the correlation between the intake amount and AUC of the metabolite concentration is a parameter which is seldom assessed, of the 14 (poly)phenols identified as showing a dose response in this study, only three (2,4-dihydroxybenzoic acid, syringic acid, and ferulic acid) have previously been shown to correlate with the amount of (poly)phenols ingested [17]. However, this was after blueberry intake, which has a different native TP profile, and further, the investigators used an enzymatic treatment prior to quantification. Therefore, all glucuronidated, sulfated, and unconjugated metabolites would have been quantified together. Unlike the previous work after blueberry ingestion, no metabolites exhibited a significant negative correlation. Other investigations into the (poly)phenol dose relationship with metabolites do not report regression analysis in this way [15,16]; such an investigation in cranberry (poly)phenols has not previously been published. Despite the fact that less than a quarter of the 60 metabolites measured showed linear dose dependence, when assessing the sum of the plasma metabolites, these were positively correlated with the amount of (poly)phenols ingested in the drink. This suggests that those 14 metabolites are key in driving the overall correlation.

Many of the metabolites assessed exhibited a very large inter-individual variability (CV up to 216%). However, each metabolite did not exhibit the same level of variability as has been discussed previously [26]. Plus, when looking at the total metabolites (sum of 60 quantified), the variability was much lower (53%), suggesting that it is not a matter of individuals poorly/effectively metabolizing poly(phenols) as a whole, but it is actually down to an individual’s ability to generate specific metabolites. There are a number of factors that will influence the metabolism and absorption. These factors may include sex, genetic polymorphisms of transporters or metabolizing enzymes, environmental influences, and likely the composition of the gut microbiome. This inter-individual variability in relation to (poly)phenols has been suggested previously [27] and may be down to the variation in the make-up of the volunteer’s gut microbiota. Recent work has shown that (poly)phenols are able to modulate the levels of gut bacteria, but it is also becoming more evident that the gut microbiota plays a very important role in (poly)phenol metabolism [28]. Thus, characterizing the native gut bacteria and investigating relationships between species of bacteria and specific metabolites will be necessary to understand this complex relationship between (poly)phenols and the gut microbiota.

The urinary recovery of the (poly)phenols was low (≤10%), although in line with urinary recoveries after other (poly)phenol-rich foods. This could be due to not capturing the entire profile of (poly)phenol metabolites and hence underestimating urinary excretion, which is feasible. Further, fecal metabolites were not considered and it is possible that a big proportion of metabolites are lost via this route, in particular the high-molecular-weight proanthocyanidins, which are not absorbed to a big extent. The method of quantification of proanthocyanidins in cranberry that we used can also explain in part the low recovery, as previously discussed [22]. Here we used the OS4-dimethylaminocinnamaldehyde (DMAC) method rather than the often used BL DMAC method. The latter overestimates (poly)phenol levels due to the use of procyanidin A2 as a standard instead of a better representation of cranberry proanthocyanidin complexity; OS DMAC includes a standardized extract comprehending larger polymers [29]. Therefore, using what we believe is a more appropriate method of quantification of (poly)phenols in cranberry juice may explain the low urinary recovery. This is specifically important in regards to this study as proanthocyanidins contribute to ~30% of the total TP amount in the cranberry beverages tested.

Although the excretion of 12 of the 60 metabolites exhibited a linear dose response, the sum of the 60 metabolites did not. Unlike the plasma metabolites, the response of the sum of the urinary metabolites seemed to plateau beyond 787 mg TP cranberry juice. Although excretion dose response is seldom investigated, similar plateaus in response to physical parameters (e.g., flow-mediated dilation) have been observed by us, in response to cranberry juice [13] and blueberry [30].

We acknowledge the limitations within this study, for example there may be metabolites that we have not measured which may impact the recovery. There are a number of compounds which escape phase I and II metabolism and undergo microbial catabolism. These compounds are transformed into small-molecular-weight compounds that are no longer polyphenol intake–specific (e.g., catechols, pyrogallols, etc.), hence making it impossible to know which are derived from the ingested (poly)phenols and which from the background diet. In addition, although the plasma was taken under the control of the analyst, most of the urine was collected under free-living conditions, and as our method of quantification was based solely on urine volume, this could have caused some error. Further, although participants were instructed regarding lifestyle and dietary adjustment prior to the study days, we had little control over this aspect. Therefore, this could have been an important factor increasing the inter-individual variation and also the differing T_max_ between cranberry juices. Although in the study we intentionally investigated the effect of cranberry juice on healthy, young, male subjects, broadening the inclusion criteria (for example, including women) or modifying the inclusion criteria (deviating from healthy participants) could have impacted our findings and could be an interesting avenue of investigation.

Taken together, we show that after consuming cranberry juice containing 409, 787, 1238, 1534, and 1910 mg total (poly)phenols, there is a linear dose-dependent increase in circulating plasma (poly)phenol metabolites, but not in urinary excretion. We note that not all individual metabolites exhibit the same effect with regard to linear regression, and we identify specific plasma metabolites that may be driving this overall effect. Further, herein we note the large inter-individual variation in the metabolic profile in response to (poly)phenol consumption and suggest that this may be in part due to inter-individual variation in the gut microbiome.

## Supplementary Materials

The following are available online at http://www.mdpi.com/2072-6643/9/3/268/s1, Table S1: Kinetic parameters after 409, 787 and 1238 mg total (poly)phenols intervention after ingestion of cranberry juice, Table S2: Kinetic parameters after 1534 and 1910 mg total (poly)phenols intervention after ingestion of cranberry juice.
