# Supplementary material for: Absorption, Metabolism and Excretion of Cranberry (Poly)phenols in Humans: A Dose Response Study and Assessment of Inter-Individual Variability
Source: Nutrients. 2017 Mar 11;9(3):268. doi: 10.3390/nu9030268 (PMC5372931; doi:10.3390/nu9030268)

**Supplementary Table 1.** Kinetic parameters after 409, 787 and 1238 mg total (poly)phenols intervention after ingestion of cranberry juice.

| | 409 mg | | | 787 mg | | | 1238 mg | | |
|---|---|---|---|---|---|---|---|---|---|
| | Cmax (nM) | Tmax (h) | AUC (nM*h) | Cmax (nM) | Tmax (h) | AUC (nM*h) | Cmax (nM) | Tmax (h) | AUC (nM*h) |
| (4R)-5-(3',4'-Dihydroxyphenyl)-gamma-valerolactone-4'-*O*-sulfate | 189 ± 80 | 4.3 ± 0.8 | 1577 ± 595 | 306 ± 60 | 3.1 ± 0.6 | 2582 ± 447 | 303 ± 53 | 6.7 ± 2.3 | 3398 ± 674 |
| 1-Methylpyrogallol-*O*-sulfate | 409 ± 119 | 8.8 ± 3.8 | 4676 ± 1150 | 538 ± 172 | 6.4 ± 3.3 | 5613 ± 1395 | 391 ± 114 | 6.3 ± 3.3 | 5279 ± 1404 |
| 2,3-dihydroxybenzoic acid | 7906 ± 2299 | 3.2 ± 0.8 | 140816 ± 38319 | 12024 ± 4055 | 2.0 ± 0.6 | 185530 ± 55774 | 10257 ± 3443 | 1.4 ± 0.3 | 175525 ± 63378 |
| 2,4-dihydroxybenzoic acid | 18 ± 4 | 6.4 ± 3.3 | 250 ± 63 | 22 ± 4 | 3.9 ± 2.5 | 280 ± 54 | 25 ± 4 | 1.4 ± 0.3 | 299 ± 57 |
| 2,5-dihydroxybenzoic acid | 419 ± 146 | 5.4 ± 1.0 | 6685 ± 2608 | 425 ± 137 | 7.3 ± 2.3 | 5888 ± 1937 | 772 ± 241 | 4.3 ± 1.1 | 11636 ± 3929 |
| 2-hydroxybenzoic acid | 658 ± 459 | 10.9 ± 3.4 | 8469 ± 5903 | 308 ± 128 | 5.3 ± 2.5 | 4857 ± 2261 | 443 ± 281 | 1.8 ± 0.5 | 6160 ± 3852 |
| 2-hydroxyhippuric acid | 9 ± 3 | 5.6 ± 0.6 | 90 ± 33 | 7 ± 2 | 4.4 ± 0.3 | 89 ± 28 | 10 ± 3 | 4.9 ± 0.6 | 118 ± 39 |
| 2-Methylpyrogallol-*O*-sulfate | 224 ± 81 | 18.4 ± 2.8 | 3095 ± 947 | 185 ± 44 | 21.8 ± 2.2 | 2821 ± 596 | 304 ± 134 | 18.0 ± 3.1 | 5145 ± 2560 |
| 3-(4-hydroxy-3-methoxyphenyl) propionic acid | 280 ± 143 | 7.2 ± 3.3 | 3242 ± 1789 | 304 ± 122 | 13.8 ± 4.0 | 3241 ± 1294 | 212 ± 105 | 8.7 ± 3.9 | 2430 ± 1259 |
| 3-(4-hydroxyphenyl) propionic acid | 312629 ± 174539 | 10.0 ± 2.8 | 3119972 ± 1428714 | 263152 ± 102592 | 7.6 ± 3.3 | 2774346 ± 1092234 | 115012 ± 39679 | 9.0 ± 3.0 | 1609708 ± 612310 |
| 3,4-dihydroxybenzaldehyde | 32 ± 10 | 5.1 ± 2.5 | 566 ± 172 | 34 ± 10 | 1.8 ± 0.8 | 618 ± 191 | 31 ± 7 | 1.0 ± 0.0 | 567 ± 153 |
| 3,4-dihydroxyphenyl acetic acid | 302 ± 88 | 8.6 ± 3.0 | 4802 ± 1490 | 476 ± 138 | 6.1 ± 2.4 | 6348 ± 2188 | 582 ± 198 | 6.0 ± 2.4 | 7443 ± 2431 |
| 3-hydroxybenzoic acid | 68 ± 22 | 0.2 ± 0.2 | 1187 ± 412 | 66 ± 23 | 0.7 ± 0.3 | 1114 ± 374 | 86 ± 35 | 4.3 ± 2.5 | 1035 ± 305 |
| 3-hydroxyhippuric acid | 41 ± 7 | 0.3 ± 0.2 | 685 ± 93 | 45 ± 7 | 0.1 ± 0.1 | 718 ± 128 | 42 ± 6 | 0.2 ± 0.1 | 698 ± 94 |
| 3-hydroxyphenyl acetic acid | 689 ± 246 | 7.6 ± 2.2 | 9780 ± 4195 | 615 ± 360 | 10.9 ± 3.4 | 9293 ± 5484 | 460 ± 115 | 9.8 ± 2.8 | 7483 ± 2018 |
| 4-hydroxybenzaldehyde | 85 ± 27 | 6.1 ± 2.4 | 1073 ± 213 | 77 ± 18 | 9.9 ± 3.6 | 1204 ± 359 | 107 ± 27 | 5.4 ± 2.5 | 1270 ± 328 |
| 4-hydroxybenzoic acid | 45 ± 12 | 7.7 ± 2.2 | 674 ± 175 | 42 ± 7 | 5.6 ± 0.9 | 605 ± 101 | 61 ± 18 | 7.1 ± 0.6 | 753 ± 198 |
| 4-hydroxyhippuric acid | 658 ± 334 | 0.0 ± 0.0 | 8670 ± 3778 | 592 ± 157 | 0.3 ± 0.2 | 6111 ± 1439 | 898 ± 318 | 0.7 ± 0.5 | 9169 ± 2315 |

| | | | | | | | | | |
|---|---|---|---|---|---|---|---|---|---|
| 4-hydroxyphenyl acetic acid | 1788 ± 732 | 3.3 ± 0.6 | 34917 ± 16343 | 1849 ± 724 | 2.9 ± 0.4 | 30711 ± 12082 | 2606 ± 1407 | 2.7 ± 0.5 | 39174 ± 18822 |
| 4-Methylcatechol-*O*-sulfate | 3160 ± 945 | 1.2 ± 0.1 | 52247 ± 15794 | 3497 ± 1192 | 1.0 ± 0.0 | 54214 ± 18798 | 2818 ± 753 | 1.0 ± 0.0 | 52455 ± 15564 |
| 4-Methylgallic-3-*O*-sulfate | 334 ± 156 | 9.1 ± 3.8 | 2314 ± 853 | 275 ± 82 | 6.1 ± 3.4 | 2188 ± 592 | 324 ± 92 | 6.4 ± 3.4 | 2440 ± 735 |
| Alfa-hydroxyhippuric acid | 2374 ± 527 | 1.0 ± 0.2 | 31185 ± 8361 | 2943 ± 587 | 1.0 ± 0.0 | 34824 ± 8717 | 4617 ± 1277 | 1.2 ± 0.1 | 46140 ± 12881 |
| Benzoic acid | 2749 ± 880 | 7.0 ± 3.3 | 55184 ± 18814 | 2169 ± 608 | 1.9 ± 0.8 | 42207 ± 12182 | 2790 ± 953 | 1.2 ± 0.1 | 52665 ± 17184 |
| Caffeic acid | 1 ± 1 | 16.7 ± 2.9 | 2 ± 1 | 1 ± 1 | 8.6 ± 3.0 | 1 ± 1 | 2 ± 2 | 3.0 ± 1.0 | 2 ± 2 |
| Caffeic Acid 3-β-D-Glucuronide | 11 ± 3 | 3.1 ± 0.8 | 68 ± 49 | 16 ± 4 | 3.8 ± 2.5 | 35 ± 10 | 34 ± 5 | 1.3 ± 0.2 | 126 ± 43 |
| Caffeic Acid 4-β-D-Glucuronide | 33 ± 6 | 7.9 ± 3.2 | 320 ± 69 | 59 ± 8 | 9.4 ± 3.7 | 380 ± 69 | 86 ± 14 | 7.1 ± 3.3 | 457 ± 79 |
| Catechol-O-sulfate | 19277 ± 3200 | 14.0 ± 4.0 | 264834 ± 45226 | 24555 ± 3775 | 6.3 ± 3.3 | 346432 ± 61239 | 17570 ± 2964 | 11.3 ± 4.0 | 258926 ± 45001 |
| Chlorogenic acid | 4 ± 2 | 4.9 ± 0.9 | 37 ± 33 | 5 ± 2 | 6.8 ± 2.3 | 40 ± 35 | 11 ± 5 | 8.3 ± 2.2 | 107 ± 59 |
| Dihydro Caffeic Acid 3-*O*-Sulfate | 1643 ± 889 | 4.7 ± 2.5 | 19433 ± 11382 | 1656 ± 1116 | 6.2 ± 2.3 | 15079 ± 10166 | 1229 ± 623 | 4.7 ± 2.5 | 16982 ± 9645 |
| Dihydro Caffeic Acid 3-*O*-β-D-Glucuronide | 90 ± 13 | 6.9 ± 0.5 | 1418 ± 206 | 84 ± 11 | 7.1 ± 0.4 | 1291 ± 207 | 99 ± 15 | 6.4 ± 0.6 | 1585 ± 268 |
| Dihydro ferulic acid 4-*O*-sulfate | 166 ± 84 | 1.8 ± 0.6 | 2334 ± 1281 | 197 ± 96 | 1.9 ± 0.6 | 2037 ± 950 | 151 ± 49 | 1.1 ± 0.3 | 1897 ± 703 |
| Dihydro Ferulic Acid 4-*O*-β-D-Glucuronide | 123 ± 25 | 6.8 ± 2.3 | 1475 ± 302 | 201 ± 59 | 8.7 ± 3.1 | 2061 ± 512 | 230 ± 64 | 6.9 ± 2.4 | 2448 ± 749 |
| Dihydro Isoferulic acid 3-*O*-ß-D-Glucuronide | 44 ± 21 | 7.4 ± 3.3 | 423 ± 158 | 23 ± 10 | 5.3 ± 2.6 | 287 ± 106 | 20 ± 4 | 10.6 ± 3.5 | 269 ± 63 |
| dihydrocaffeic acid | 91 ± 31 | 1.8 ± 0.4 | 1254 ± 346 | 93 ± 32 | 2.1 ± 0.3 | 1451 ± 535 | 91 ± 31 | 2.1 ± 0.3 | 1436 ± 529 |
| DihydroIsoferulic acid 3-*O*-sulfate | 90 ± 41 | 2.1 ± 0.8 | 1154 ± 646 | 97 ± 42 | 2.1 ± 0.8 | 859 ± 449 | 83 ± 20 | 1.2 ± 0.1 | 748 ± 243 |
| Ferulic acid 4-*O*-glucuronide | 105 ± 13 | 5.9 ± 2.4 | 760 ± 109 | 165 ± 29 | 9.1 ± 3.8 | 1062 ± 181 | 266 ± 39 | 9.0 ± 3.8 | 1394 ± 184 |
| Ferulic Acid 4-*O*-Sulfate | 1209 ± 470 | 4.4 ± 2.5 | 8749 ± 4547 | 2268 ± 794 | 1.2 ± 0.1 | 11484 ± 5522 | 2758 ± 883 | 1.6 ± 0.3 | 13080 ± 5235 |
| Hippuric acid | 54624 ± 15425 | 5.7 ± 2.5 | 862928 ± 230484 | 42926 ± 12282 | 8.1 ± 3.1 | 758245 ± 223886 | 63012 ± 20922 | 4.8 ± 2.4 | 1052242 ± 327861 |
| Homovanillic acid | 491 ± 173 | 16.0 ± 3.2 | 8797 ± 3227 | 511 ± 165 | 22.2 ± 1.8 | 9005 ± 2913 | 797 ± 349 | 19.7 ± 2.9 | 11313 ± 3885 |
| Homovanillic acid sulfate | 33 ± 14 | 3.8 ± 2.5 | 512 ± 218 | 30 ± 10 | 1.1 ± 0.2 | 522 ± 178 | 34 ± 10 | 1.2 ± 0.1 | 635 ± 199 |

| | | | | | | | | | |
|---|---|---|---|---|---|---|---|---|---|
| Isoferulic acid | 5530 ± 2788 | 7.8 ± 3.2 | 87542 ± 52916 | 4592 ± 2251 | 7.7 ± 3.2 | 79971 ± 46457 | 4618 ± 1998 | 10.9 ± 3.3 | 58885 ± 23712 |
| Isoferulic acid 3-*O*-sulfate | 35 ± 6 | 4.7 ± 2.5 | 452 ± 87 | 49 ± 6 | 7.1 ± 3.3 | 529 ± 90 | 69 ± 15 | 6.9 ± 3.4 | 621 ± 117 |
| Isoferulic Acid 3-*O*-β-D-Glucuronide | 555 ± 120 | 3.7 ± 2.5 | 2893 ± 575 | 387 ± 95 | 3.6 ± 2.6 | 2534 ± 538 | 425 ± 130 | 1.2 ± 0.1 | 2251 ± 641 |
| Isovanillic acid | 231 ± 52 | 2.1 ± 0.8 | 3171 ± 596 | 220 ± 44 | 3.2 ± 0.8 | 3087 ± 558 | 235 ± 66 | 4.7 ± 2.5 | 3152 ± 562 |
| kaempferol | 53 ± 14 | 1.1 ± 0.2 | 1021 ± 300 | 59 ± 18 | 1.8 ± 0.4 | 1035 ± 305 | 44 ± 14 | 1.4 ± 0.3 | 852 ± 246 |
| Kaempferol-3-glucuronide | 11 ± 2 | 1.9 ± 0.8 | 101 ± 9 | 13 ± 1 | 2.1 ± 0.4 | 118 ± 12 | 14 ± 2 | 2.3 ± 0.4 | 139 ± 16 |
| *m*-Coumaric acid | 16 ± 5 | 3.4 ± 0.9 | 183 ± 71 | 29 ± 14 | 1.2 ± 0.3 | 322 ± 150 | 16 ± 6 | 3.9 ± 2.5 | 171 ± 67 |
| *o*-Coumaric acid | 6 ± 1 | 2.9 ± 1.1 | 103 ± 21 | 6 ± 1 | 6.2 ± 2.4 | 99 ± 13 | 5 ± 1 | 3.8 ± 1.0 | 97 ± 15 |
| *p*-Coumaric acid | 71 ± 31 | 7.1 ± 3.2 | 188 ± 103 | 131 ± 51 | 7.3 ± 3.2 | 320 ± 133 | 240 ± 141 | 6.6 ± 3.3 | 530 ± 300 |
| Phenylacetic acid | 8110 ± 2449 | 5.7 ± 2.4 | 118624 ± 37549 | 8304 ± 1886 | 5.1 ± 2.4 | 123805 ± 28913 | 7929 ± 2021 | 4.2 ± 2.5 | 118686 ± 29004 |
| Protocatechuic acid | 96 ± 39 | 6.0 ± 2.4 | 1181 ± 475 | 109 ± 45 | 10.2 ± 3.5 | 1196 ± 442 | 116 ± 52 | 8.9 ± 3.0 | 1142 ± 469 |
| Pyrogallol-*O*-1-sulfate | 202 ± 70 | 1.6 ± 0.9 | 3254 ± 1131 | 199 ± 79 | 5.1 ± 2.5 | 3393 ± 1338 | 245 ± 113 | 3.2 ± 2.6 | 4074 ± 1753 |
| Pyrogallol-*O*-2-sulfate | 315 ± 116 | 4.2 ± 0.7 | 2428 ± 958 | 339 ± 123 | 3.8 ± 2.6 | 3007 ± 1095 | 262 ± 131 | 3.9 ± 2.6 | 2681 ± 1388 |
| Quercetin-glucuronide | 68 ± 16 | 6.0 ± 3.4 | 467 ± 81 | 156 ± 35 | 8.3 ± 3.9 | 910 ± 162 | 248 ± 45 | 8.9 ± 3.8 | 1420 ± 256 |
| Sinapic acid | 29 ± 7 | 7.0 ± 3.3 | 287 ± 58 | 46 ± 11 | 4.1 ± 2.6 | 358 ± 84 | 64 ± 20 | 4.0 ± 2.6 | 399 ± 115 |
| Syringic acid | 0 ± 0 | 3.9 ± 2.5 | 0 ± 0 | 8 ± 6 | 1.3 ± 0.2 | 21 ± 20 | 9 ± 9 | 1.6 ± 0.3 | 18 ± 17 |
| *t*-Cinnamic acid | 98 ± 18 | 6.4 ± 2.4 | 1791 ± 363 | 123 ± 34 | 4.9 ± 2.5 | 1888 ± 399 | 90 ± 17 | 2.0 ± 0.4 | 1642 ± 355 |
| *t*-Ferulic acid | 25 ± 9 | 9.9 ± 3.6 | 82 ± 29 | 47 ± 12 | 6.8 ± 3.3 | 148 ± 51 | 74 ± 25 | 6.1 ± 3.4 | 190 ± 52 |
| Vanillic acid | 311 ± 84 | 4.1 ± 2.5 | 3586 ± 1161 | 410 ± 115 | 4.0 ± 2.5 | 3700 ± 1070 | 667 ± 223 | 3.9 ± 2.5 | 3540 ± 960 |
| Vanillic acid-4-*O*-sulfate | 814 ± 302 | 4.3 ± 2.6 | 10775 ± 4640 | 1054 ± 274 | 4.1 ± 2.5 | 11791 ± 3372 | 1355 ± 337 | 6.4 ± 3.3 | 14072 ± 4824 |

**Supplementary Table 2.** Kinetic parameters after 1534 and 1910 mg total (poly)phenols intervention after ingestion of cranberry juice.

| | 1534 mg | | | 1910 mg | | |
|---|---|---|---|---|---|---|
| | Cmax (nM) | Tmax (h) | AUC (nM*h) | Cmax (nM) | Tmax (h) | AUC (nM*h) |
| (4R)-5-(3',4'-Dihydroxyphenyl)-gamma-valerolactone-4'-*O*-sulfate | 430 ± 111 | 6.0 ± 2.3 | 4098 ± 930 | 683 ± 203 | 6.2 ± 2.3 | 5614 ± 1370 |
| 1-Methylpyrogallol-*O*-sulfate | 561 ± 132 | 3.7 ± 2.5 | 5546 ± 1311 | 308 ± 63 | 6.8 ± 3.3 | 4647 ± 828 |
| 2,3-dihydroxybenzoic acid | 11866 ± 3403 | 1.3 ± 0.3 | 161941 ± 43351 | 8936 ± 2516 | 1.6 ± 0.3 | 162144 ± 46199 |
| 2,4-dihydroxybenzoic acid | 30 ± 7 | 1.1 ± 0.1 | 354 ± 70 | 33 ± 7 | 1.3 ± 0.3 | 367 ± 60 |
| 2,5-dihydroxybenzoic acid | 834 ± 254 | 5.4 ± 1.1 | 11991 ± 3885 | 1459 ± 622 | 5.6 ± 0.8 | 19318 ± 7969 |
| 2-hydroxybenzoic acid | 512 ± 266 | 2.3 ± 0.9 | 7207 ± 3838 | 628 ± 316 | 1.3 ± 0.4 | 7717 ± 4048 |
| 2-hydroxyhippuric acid | 13 ± 5 | 5.8 ± 0.5 | 144 ± 61 | 202 ± 192 | 5.6 ± 0.4 | 1684 ± 1554 |
| 2-Methylpyrogallol-*O*-sulfate | 167 ± 30 | 15.7 ± 3.3 | 2385 ± 558 | 312 ± 126 | 15.6 ± 3.4 | 4825 ± 1565 |
| 3-(4-hydroxy-3-methoxyphenyl) propionic acid | 217 ± 80 | 11.7 ± 3.9 | 3403 ± 1606 | 102 ± 40 | 9.6 ± 3.6 | 1605 ± 612 |
| 3-(4-hydroxyphenyl) propionic acid | 267255 ± 117900 | 10.6 ± 3.5 | 2708985 ± 1049438 | 390606 ± 270423 | 11.0 ± 3.4 | 2467256 ± 1001474 |
| 3,4-dihydroxybenzaldehyde | 29 ± 9 | 3.6 ± 2.6 | 541 ± 187 | 29 ± 8 | 2.1 ± 0.8 | 520 ± 143 |
| 3,4-dihydroxyphenyl acetic acid | 635 ± 170 | 6.0 ± 2.4 | 8707 ± 2929 | 824 ± 310 | 4.9 ± 2.5 | 13366 ± 5976 |
| 3-hydroxybenzoic acid | 76 ± 22 | 3.2 ± 2.6 | 1234 ± 398 | 96 ± 31 | 1.8 ± 0.7 | 1619 ± 615 |
| 3-hydroxyhippuric acid | 41 ± 7 | 1.1 ± 0.5 | 678 ± 139 | 49 ± 10 | 0.3 ± 0.2 | 872 ± 159 |
| 3-hydroxyphenyl acetic acid | 333 ± 72 | 17.1 ± 3.5 | 5524 ± 1508 | 486 ± 130 | 13.4 ± 3.4 | 8898 ± 2664 |
| 4-hydroxybenzaldehyde | 88 ± 20 | 6.3 ± 3.3 | 1220 ± 271 | 96 ± 23 | 6.9 ± 3.3 | 1427 ± 413 |
| 4-hydroxybenzoic acid | 65 ± 18 | 7.3 ± 0.3 | 718 ± 145 | 103 ± 37 | 9.3 ± 1.9 | 1186 ± 511 |
| 4-hydroxyhippuric acid | 624 ± 205 | 0.8 ± 0.3 | 7725 ± 2465 | 860 ± 288 | 0.9 ± 0.5 | 10507 ± 3414 |
| 4-hydroxyphenyl acetic acid | 1702 ± 657 | 2.9 ± 0.4 | 26021 ± 9842 | 3831 ± 2588 | 2.9 ± 0.5 | 64387 ± 41410 |

| | | | | | | |
|---|---|---|---|---|---|---|
| 4-Methylcatechol-*O*-sulfate | 4951 ± 1503 | 1.0 ± 0.0 | 76095 ± 24228 | 4067 ± 1192 | 1.0 ± 0.0 | 71307 ± 22629 |
| 4-Methylgallic-3-*O*-sulfate | 397 ± 113 | 4.0 ± 2.6 | 3387 ± 1074 | 472 ± 139 | 6.6 ± 3.4 | 3287 ± 988 |
| Alfa-hydroxyhippuric acid | 4580 ± 905 | 1.6 ± 0.6 | 41567 ± 9650 | 4991 ± 1101 | 1.4 ± 0.3 | 61145 ± 24179 |
| Benzoic acid | 2327 ± 564 | 3.6 ± 2.6 | 42378 ± 10771 | 4393 ± 1986 | 1.0 ± 0.0 | 82430 ± 37764 |
| Caffeic acid | 12 ± 6 | 7.7 ± 3.2 | 27 ± 12 | 8 ± 4 | 8.0 ± 3.1 | 9 ± 5 |
| Caffeic Acid 3-β-D-Glucuronide | 36 ± 7 | 1.4 ± 0.3 | 94 ± 25 | 45 ± 11 | 2.7 ± 0.7 | 125 ± 35 |
| Caffeic Acid 4-β-D-Glucuronide | 110 ± 24 | 9.4 ± 3.7 | 531 ± 97 | 119 ± 15 | 4.8 ± 2.6 | 562 ± 65 |
| Catechol-O-sulfate | 20935 ± 3471 | 6.9 ± 3.3 | 295335 ± 52160 | 22662 ± 2933 | 4.1 ± 2.5 | 323621 ± 40578 |
| Chlorogenic acid | 9 ± 4 | 11.0 ± 3.4 | 63 ± 40 | 5 ± 2 | 8.3 ± 3.1 | 55 ± 43 |
| Dihydro Caffeic Acid 3-*O*-Sulfate | 1665 ± 586 | 7.0 ± 3.3 | 14345 ± 6067 | 2181 ± 880 | 2.9 ± 0.5 | 25980 ± 10353 |
| Dihydro Caffeic Acid 3-*O*-β-D-Glucuronide | 91 ± 11 | 6.4 ± 0.4 | 1385 ± 211 | 110 ± 13 | 6.9 ± 0.4 | 1797 ± 251 |
| Dihydro ferulic acid 4-*O*-sulfate | 124 ± 41 | 1.0 ± 0.3 | 1771 ± 656 | 179 ± 65 | 4.6 ± 2.5 | 2533 ± 941 |
| Dihydro Ferulic Acid 4-*O*-β-D-Glucuronide | 150 ± 23 | 7.1 ± 2.3 | 2062 ± 415 | 581 ± 469 | 9.9 ± 2.8 | 7523 ± 5927 |
| Dihydro Isoferulic acid 3-*O*-ß-D-Glucuronide | 24 ± 4 | 5.9 ± 3.4 | 284 ± 52 | 69 ± 42 | 9.3 ± 3.7 | 849 ± 513 |
| dihydrocaffeic acid | 97 ± 32 | 4.2 ± 2.5 | 1521 ± 464 | 188 ± 104 | 2.6 ± 0.4 | 3169 ± 1620 |
| DihydroIsoferulic acid 3-*O*-sulfate | 112 ± 32 | 1.2 ± 0.1 | 998 ± 408 | 149 ± 35 | 2.8 ± 0.6 | 1227 ± 357 |
| Ferulic acid 4-*O*-glucuronide | 312 ± 65 | 8.7 ± 3.8 | 1469 ± 242 | 364 ± 46 | 12.2 ± 3.7 | 1955 ± 295 |
| Ferulic Acid 4-*O*-Sulfate | 3711 ± 1184 | 1.3 ± 0.2 | 13938 ± 5069 | 4865 ± 1508 | 1.4 ± 0.3 | 15031 ± 4415 |
| Hippuric acid | 51925 ± 15250 | 7.2 ± 3.3 | 816882 ± 244519 | 90567 ± 40359 | 8.9 ± 2.9 | 1324622 ± 469127 |
| Homovanillic acid | 675 ± 230 | 19.4 ± 3.0 | 12193 ± 4341 | 1756 ± 1003 | 15.6 ± 3.4 | 28983 ± 15511 |
| Homovanillic acid sulfate | 38 ± 12 | 1.2 ± 0.1 | 633 ± 212 | 52 ± 15 | 1.2 ± 0.4 | 964 ± 313 |
| Isoferulic acid | 4238 ± 1440 | 10.4 ± 3.5 | 54622 ± 20556 | 7545 ± 3462 | 14.4 ± 3.8 | 129174 ± 70072 |
| Isoferulic acid 3-*O*-sulfate | 63 ± 10 | 9.2 ± 3.8 | 564 ± 94 | 76 ± 14 | 5.6 ± 2.4 | 622 ± 87 |
| Isoferulic Acid 3-*O*-β-D-Glucuronide | 233 ± 120 | 1.2 ± 0.1 | 1175 ± 574 | 441 ± 119 | 1.3 ± 0.3 | 3339 ± 972 |
| Isovanillic acid | 232 ± 56 | 4.4 ± 2.6 | 2850 ± 519 | 340 ± 110 | 7.2 ± 3.3 | 4054 ± 1153 |

| | | | | | | |
|---|---|---|---|---|---|---|
| kaempferol | 50 ± 15 | 1.3 ± 0.2 | 973 ± 283 | 53 ± 15 | 2.1 ± 0.5 | 918 ± 294 |
| Kaempferol-3-glucuronide | 20 ± 3 | 1.2 ± 0.2 | 145 ± 21 | 18 ± 2 | 2.4 ± 0.4 | 185 ± 30 |
| *m*-Coumaric acid | 12 ± 4 | 2.1 ± 0.6 | 165 ± 67 | 13 ± 4 | 3.4 ± 2.6 | 187 ± 79 |
| *o*-Coumaric acid | 6 ± 1 | 4.4 ± 0.9 | 97 ± 17 | 7 ± 1 | 2.3 ± 0.6 | 110 ± 16 |
| *p*-Coumaric acid | 251 ± 108 | 1.8 ± 0.6 | 561 ± 245 | 364 ± 197 | 7.4 ± 3.2 | 861 ± 472 |
| Phenylacetic acid | 8054 ± 2116 | 4.3 ± 2.6 | 124585 ± 33830 | 10349 ± 3761 | 1.8 ± 0.4 | 141391 ± 40701 |
| Protocatechuic acid | 92 ± 32 | 10.1 ± 3.6 | 880 ± 310 | 136 ± 60 | 11.8 ± 3.1 | 1836 ± 864 |
| Pyrogallol-*O*-1-sulfate | 211 ± 80 | 6.9 ± 3.3 | 3348 ± 1208 | 198 ± 63 | 2.6 ± 0.7 | 3280 ± 1002 |
| Pyrogallol-*O*-2-sulfate | 406 ± 132 | 8.0 ± 3.1 | 3192 ± 1163 | 191 ± 54 | 8.0 ± 3.1 | 1664 ± 424 |
| Quercetin-glucuronide | 342 ± 74 | 10.9 ± 4.1 | 1841 ± 362 | 416 ± 112 | 11.7 ± 3.9 | 2150 ± 352 |
| Sinapic acid | 78 ± 22 | 4.1 ± 2.6 | 404 ± 96 | 104 ± 30 | 6.0 ± 3.4 | 674 ± 252 |
| Syringic acid | 21 ± 8 | 1.3 ± 0.2 | 49 ± 22 | 23 ± 12 | 1.4 ± 0.3 | 78 ± 42 |
| *t*-Cinnamic acid | 96 ± 17 | 2.2 ± 0.8 | 1748 ± 344 | 116 ± 18 | 4.6 ± 2.5 | 1964 ± 382 |
| *t*-Ferulic acid | 82 ± 27 | 9.0 ± 3.8 | 238 ± 68 | 130 ± 43 | 10.2 ± 3.5 | 682 ± 427 |
| Vanillic acid | 726 ± 229 | 1.0 ± 0.2 | 3891 ± 1053 | 952 ± 305 | 1.9 ± 0.6 | 6321 ± 2609 |
| Vanillic acid-4-*O*-sulfate | 1528 ± 416 | 1.1 ± 0.2 | 14800 ± 4781 | 1576 ± 368 | 1.3 ± 0.3 | 20293 ± 6818 |

Supplementary Figure 1

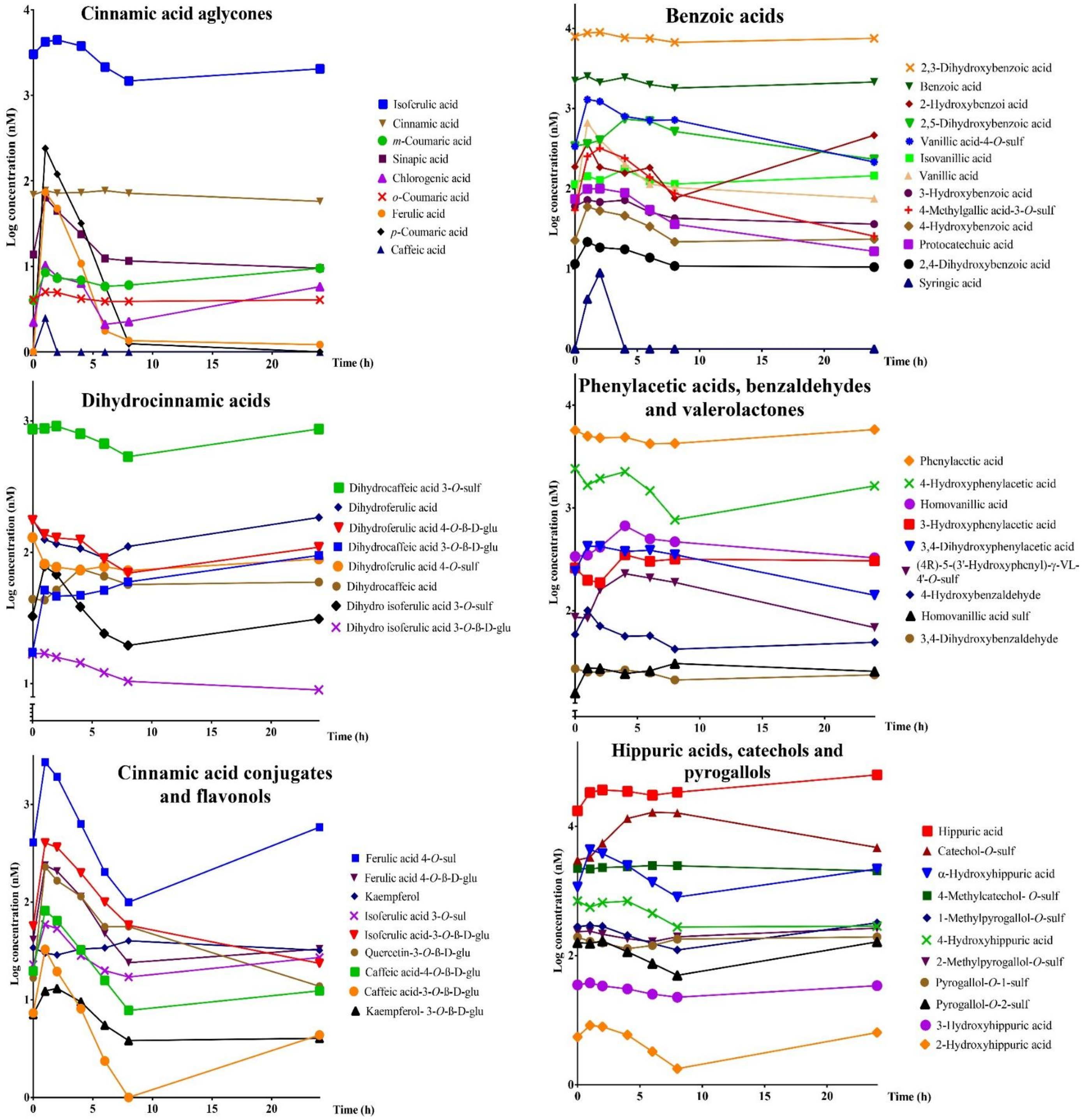

Supplement: Supplementary file 1 [file nutrients-09-00268-s001.pdf]
